# Patterns and resectability of colorectal cancer recurrences: outcome study within the COLOFOL trial

**DOI:** 10.1093/bjsopen/zrab067

**Published:** 2021-07-26

**Authors:** P Hansdotter, P Scherman, S H Petersen, M Mikalonis, E Holmberg, M Rizell, P Naredi, I Syk

**Affiliations:** Department of Surgery, Skåne University Hospital, Malmö, Sweden; Institute of Clinical Sciences Malmö, Section of Surgery, Lund University, Lund, Sweden; Department of Surgery, Institute of Clinical Sciences, University of Gothenburg, Gothenburg, Sweden; Department of Surgery, Helsingborg Hospital, Helsingborg, Sweden; Department of Paediatrics and Adolescent Medicine, Section of Paediatric Haematology and Oncology, Rigshospitalet, Copenhagen, Denmark; Department of Surgery, Aalborg Hospital, Aalborg, Denmark; Department of Oncology, Institute of Clinical Sciences, University of Gothenburg, Gothenburg, Sweden; Department of Surgery, Institute of Clinical Sciences, University of Gothenburg, Gothenburg, Sweden; Transplant Institute, Sahlgrenska University Hospital, Gothenburg, Sweden; Department of Surgery, Institute of Clinical Sciences, University of Gothenburg, Gothenburg, Sweden; Department of Surgery, Sahlgrenska University Hospital, Gothenburg, Sweden; Department of Surgery, Skåne University Hospital, Malmö, Sweden; Institute of Clinical Sciences Malmö, Section of Surgery, Lund University, Lund, Sweden

## Abstract

**Background:**

Improvements in surgery, imaging, adjuvant treatment, and management of metastatic disease have led to modification of previous approaches regarding the risk of recurrence and prognosis in colorectal cancer. The aims of this study were to map patterns, risk factors, and the possibility of curative treatment of recurrent colorectal cancer in a multimodal setting.

**Methods:**

This was a cohort study based on the COLOFOL trial population of patients who underwent radical resection of stage II or III colorectal cancer. The medical files of all patients with recurrence within 5 years after resection of the primary tumour were scrutinized. Follow-up time was 5 years after the first recurrence. Primary endpoints were cumulative incidence, site, timing, and risk factors for recurrence, and rate of potentially curative treatment. A secondary endpoint was survival.

**Results:**

Of 2442 patients, 471 developed recurrences. The 5-year cumulative incidence was 21.4 (95 per cent c.i. 19.5 to 23.3) per cent. The median time to detection was 1.1 years after surgery and 87.3 per cent were detected within 3 years. Some 98.2 per cent of patients who had potentially curative treatment were assessed by a multidisciplinary tumour board. A total of 47.8 per cent of the recurrences were potentially curatively treated. The 5-year overall survival rate after detection was 32.0 (95 per cent c.i. 27.9 to 36.3) per cent for all patients with recurrence, 58.6 (51.9 to 64.7) per cent in the potentially curatively treated group and 7.7 (4.8 to 11.5) per cent in the palliatively treated group.

**Conclusion:**

Time to recurrence was similar to previous results, whereas the 21.4 per cent risk of recurrence was somewhat lower. The high proportion of patients who received potentially curative treatment, linked to a 5-year overall survival rate of 58.6 per cent, indicates that it is possible to achieve good results in recurrent colorectal cancer following multidisciplinary assessment.

## Teaser

The 5-year cumulative recurrence rate after radical surgery for stage II–III colorectal cancer was 21.4 per cent. A total of 47.8 per cent of recurrences could be treated potentially curatively, which was associated with a 5-year overall survival rate of 58.6 per cent. Treatment of recurrences confined to the liver was most likely to be potentially curative (75.7 per cent). A high proportion of recurrences confined to the lungs and peritoneum were also treated with curative intent.

## Introduction

Colorectal cancer is the second most common cause of cancer-related mortality worldwide^1^. Following surgery with curative intent, some 10–35 per cent of patients develop metachronous metastases^2–8^. This range of reported recurrences reflects changes in the accuracy of preoperative staging, as well as different postoperative imaging between centres and time periods. These imaging techniques have improved markedly over the past two decades enabling earlier detection of metastases, making preoperative staging and postoperative surveillance examinations more accurate. Adjuvant chemotherapy has been adopted widely to prevent some recurrences^8–10^. Considering all these factors, earlier estimates of the incidence of metachronous metastases and the likelihood of offering further treatment designed to achieve cure may now be inaccurate.

Follow-up programmes designed to detect recurrences that are possible to treat with curative intent are standard nowadays. Although large retrospective studies failed to prove any survival benefit from such programmes^11^, small randomized trials that followed had some positive results, and subsequent systematic reviews and meta-analysis^12^^,^^13^ indicated survival benefit after more frequent examinations. Later large, randomized trials, such as COLOFOL^14^, GILDA^15^ and FACS^16^, could not establish a survival benefit from more frequent follow-up, although more recurrences in the high-frequency follow-up arm could be treated with curative intent. A Cochrane meta-analysis^17^ came to the same conclusion. The optimal design of a follow-up programme after curative resection for colorectal cancer is still unclear and proof of benefit resulting from intensive follow-up is lacking .

Whether a follow-up programme will lead to survival benefit depends on its ability to detect asymptomatic recurrences, and treat them with curative intent. The proportion of recurrences treated with curative intent varies markedly^6^^,^^18^^,^^19^, reflecting differences in follow-up routines, multimodal treatment algorithms, and selection criteria for management of metastases. To optimize and individualize adjuvant therapies and design a surveillance programme with a positive effect on survival, it is important to know the risk factors and pattern of recurrences in a population of patients with colorectal cancer managed with modern multimodal treatment. This should include an understanding of patterns of recurrence amenable to treatment with curative intent. The aim of this study was to map the pattern of, and risk factors for, recurrences in a well defined population of patients with colorectal cancer who had undergone a multimodal treatment approach including curative surgery, and to evaluate the proportion of recurrences possible to treat with curative intent, based on the COLOFOL study cohort. Primary endpoints were the cumulative incidence, timing, and site of recurrence, risk factors for recurrence, and rate of potentially curatively treatment. Secondary endpoints were 5-year overall survival (OS) depending on recurrence site and mode of detection.

## Methods

All patients in the study cohort were identified in the COLOFOL trial population. Detailed information on the COLOFOL trial study design and population has been reported previously^14^^,^^20^. This study was not included in the original study plan. In summary, the COLOFOL trial enrolled patients who underwent radical surgery for stage II or III sporadic colorectal cancer between 2006 and 2010 at 24 sites in Sweden (15), Denmark (8), and Uruguay (1). Patients had to be aged 18–75 years with a life expectancy exceeding 2 years based on co-morbidity. The objective of the study was to compare overall and cancer-specific mortality according to follow-up regimen. Patients were randomized to either high- or low-intensity follow-up, with contrast-enhanced multislice CT of the abdomen and thorax at certified centres, along with measurement of serum levels of carcinoembryonic antigen (CEA). Examinations were performed 6, 12, 18, 24, and 36 months after surgery (high-intensity group; 1253 patients) or at 12 and 36 months after surgery (low-intensity group; 1256 patients). A colonoscopy was required in the perioperative period to verify a clean colon, whereas further endoscopies were optional. All patients had to personally provide written consent before embarking in the study. The trial was approved by the ethical committee of Uppsala University (2004: M-453) in Sweden, and Copenhagen and Frederiksberg Scientific committee (KF 01–194/04) in Denmark.

All patients were followed prospectively for 5 years after resection of the primary tumour. For the present study, all patients registered with recurrences within 5 years after primary surgery in Sweden and Denmark were identified. The figures presented reflect recurrence rates within 5 years; late recurrences beyond that time were not covered. All medical files were scrutinized for detailed information on time point and type of recurrence, and means of detection and treatment, including both surgical and medical treatment for each recurrence. Patients from Uruguay were not included. Mortality was checked through the population registries in Denmark and Sweden. Follow-up time after first recurrence was 5 years in all but one patient.

Data collected included: age, sex, date of detection of the recurrence, location of recurrence, method of detection, surgical and medical treatment of the recurrence including adjuvant chemotherapy, and aim of treatment (curative or palliative). The same data were collected for any second- and third-line treatments, if given. Data collected from the time of primary surgery were: BMI, concurrent diseases (lung disease, diabetes, history of myocardial infarction), smoking and alcohol habits, CEA level, date of surgery, site of tumour, adjuvant radiotherapy and/or chemotherapy, urgency of operation (acute or elective), blood transfusion, postoperative complications, and detailed information in the pathology report.

Recurrence in mesenteric lymph nodes was defined as a local recurrence, whereas any recurrence in distant lymph nodes, including inguinal or para-aortic nodes, was defined as metastasis (M1). Anastomotic recurrences as well as retroperitoneal recurrences in the operative field of the bowel resection were considered local recurrences, whereas any other recurrence involving the peritoneum and/or omentum was defined as a peritoneal recurrence. Potentially curative treatment was defined as fulfilled resection or ablative treatment judged clinically as radical.

Time was measured from date of surgery to the first of the following events during 5-year follow-up: recurrence, death, or end of follow-up. The cumulative incidence of recurrence was computed using a competing-risk method, with death as a competing event and end of follow-up as a censoring event. Cumulative incidence reflects the probability of developing a recurrence during the time period, which also can be described as absolute risk during this interval. To facilitate readability, the term risk was used to describe the cumulative incidence during the study period.

### Statistical analysis

All statistical analyses were carried out with Stata^®^ version 16.1 (StataCorp, College Station, TX, USA). Figures for cumulative incidence in the presence of competing events were generated by means of the macro stcompet for Stata^®^^21^. Both the cumulative incidence of recurrence and cumulative incidence of competing event (death) were calculated with 95 per cent confidence interval. Hazard ratios (HRs) were calculated by cause-specific univariable and multivariable Cox proportional hazards regression to reflect the relative risk of recurrence between groups. In these analyses, death was a censoring event.

Secondary endpoints were: 5-year OS according to recurrence site and mode of detection. OS was computed using the Kaplan–Meier method and group comparisons were made by univariable and multivariable Cox proportional hazards regression.

The proportional hazards assumption was tested with Schoenfeld’s residuals. When the assumption was violated (*P* < 0.050), the follow-up period was divided at 1 year into two intervals to achieve proportional hazards. All collected variables were included in the multivariable Cox regression analyses, and retained in the model if they were independently statistically significant or had *P* < 0.200 and a confounding effect (affected other HRs by more than 10 per cent). CEA was omitted from the analysis because there were too many missing values. *P* < 0.050 was considered statistically significant.

## Results

A total of 2456 patients were randomized in the COLOFOL trial in Sweden and Denmark, of whom 14 were excluded as they did not fulfil the inclusion criteria or lacked information on recurrences. The present study involved 2442 patients, of whom 494 were registered with recurrences within 5 years after surgery. Following medical record review, 23 patients were reclassified without recurrence as no recurrences could be confirmed. Of these, eight patients were diagnosed with a new primary colorectal cancer, seven with primary lung cancer, one with a suspected mesenteric metastasis that proved to be benign, and one with primary ovarian cancer; in six patients, no obvious explanation could be established. A total of 471 patients were therefore confirmed to have recurrent disease and constituted the cohort of patients with recurrences.

### Risk, site, and timing of recurrences

The total cumulative risk of recurrence was 21.4 (95 per cent c.i. 19.5 to 23.3) per cent. It was 13.4 (11.4 to 15.6) per cent in stage II and 30.7 (27.6 to 33.9) per cent in stage III disease (*Table 1*). No difference in risk of recurrence was noted between the Swedish and Danish cohorts (data not shown).

**Table 1:** Recurrences within 5 years after radical resection for stage II or III colorectal cancer, stratified by tumour stage and primary tumour location

	No. of patients	Recurrences				
		All	Liver only	Lung only	Other location	Multiple locations
**Overall**	2442	471 (21.4)	148 (6.5)	89 (4.0)	91 (4.6)	143 (6.3)
**Stage II**	1315	161 (13.4)	64 (5.4)	29 (2.4)	30 (2.6)	37 (3.1)
T3 N0	1144	121 (11.5)	50 (4.7)	25 (2.3)	18 (1.7)	28 (2.8)
T4 N0	169	39 (25.7)	14 (9.7)	4 (2.4)	12 (8.3)	9 (5.3)
Missing	2	1	0	1	0	0
**Stage III**	1127	310 (30.7)	84 (7.8)	59 (5.8)	61 (7.1)	106 (10.1)
Total						
T1–3 N1	657	132 (23.4)	40 (6.4)	33 (5.2)	24 (5.5)	35 (6.3)
T1–3 N2	289	109 (41.8)	32 (11.2)	21 (9.2)	19 (8.5)	37 (12.9)
T4 N1	83	24 (31.5)	9 (12.8)	0	8 (10.3)	7 (8.4)
T4 N2	94	45 (48.2)	3 (3.2)	5 (5.3)	10 (10.7)	27 (29.0)
Missing	4	0	0	0	0	0
**Location**						
Colon	1585	264 (18.3)	90 (6.1)	21 (1.5)	59 (4.4)	94 (6.3)
Rectum	857	207 (27.4)	58 (7.4)	68 (8.6)	32 (5.1)	49 (6.3)

Values in parentheses are percentage cumulative risks at 5 years.

**Fig. 1:**
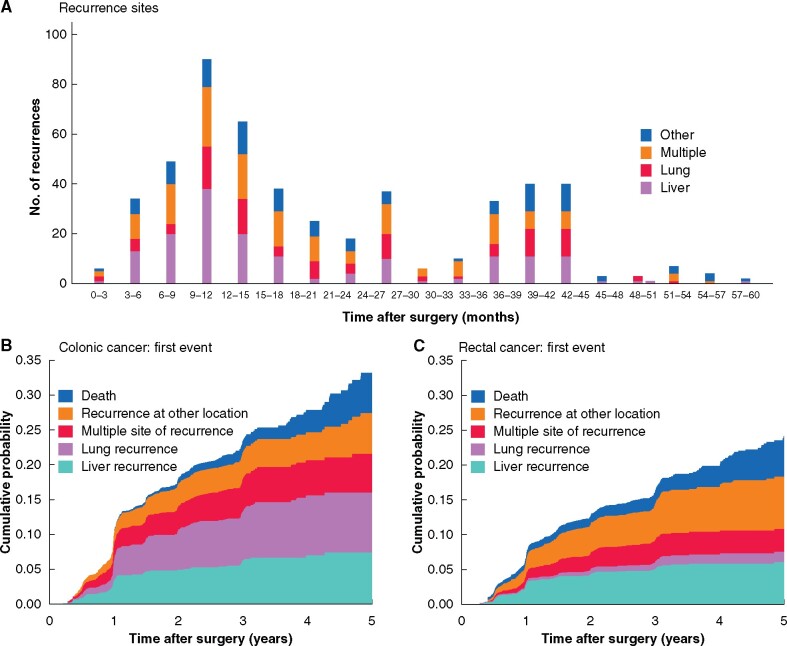
Time of detection of recurrences within 5 years following radical resection of stage II or III colorectal cancer, stratified by site of recurrence. A) Stratified by 3-month periods after randomization; B) cumulative incidence for colonic cancer (including mortality as competing risk), and C) cumulative incidence for rectal cancer (including mortality as competing risk).

In total, 328 patients (69.6 per cent) developed a first recurrence at a single site, whereas 143 (30.4 per cent) developed recurrences at multiple sites. The most common site of first recurrence was liver (9.6 per cent), followed by lung (6.8 per cent). Detailed information on site of recurrences is shown in *Table 2*. The median time to detection of recurrences was 1.1 years, and 87.3 per cent of the recurrences were detected within 3 years. The distribution, timing of detection, and cumulative incidence of all recurrences are presented in *Fig. 1a–**c*.

**Table 2:** First recurrence within 5 years in patients who underwent primary radical surgery for stage II or III colorectal cancer, stratified by location

Site of metastases	Liver	Lung	Peritoneum	Lymph nodes	Local	Other
Liver	148 (6.1)	23 (0.9)	5 (0.2)	11 (0.4)	6 (0.2)	1 (0.0)
Lung	23 (0.9)	89 (3.6)	0 (0)	8 (0.3)	7 (0.3)	1 (0.0)
Peritoneum	5 (0.2)	0 (0)	23 (0.9)	2 (0.1)	16 (0.7)	0 (0)
Lymph nodes	11 (0.4)	8 (0.3)	2 (0.1)	25 (1.0)	5 (0.2)	2 (0.1)
Local recurrence	6 (0.2)	7 (0.3)	16 (0.7)	5 (0.2)	38 (1.6)	1 (0.0)
Other	1 (0.0)	1 (0.0)	0 (0 )	2 (0.1)	1 (0.0)	5 (0.2)
≥3 sites	41 (1.7)	39 (1.6)	26 (1.1)	33 (1.4)	21 (0.9)	21 (0.9)
Total	235 (9.6)	167 (6.8)	72 (3.0)	86 (3.5)	94 (3.8)	31 (1.3)

Values in parentheses are percentages.

### Risk factors for recurrence

A higher risk of recurrence was noted in rectal compared with colonic cancer: 27.4 (95 per cent c.i. 23.9 to 31.2) and 18.3 (16.3 to 20.5) per cent respectively (*Table 3*); there was a significantly higher risk of pulmonary metastases in rectal cancer, at 12.5 (10.3 to15.0 ) per cent compared with 4.5 per cent (3.5 to 5.7 ) per cent in colonic cancer (*Fig. 1b,c*). No difference was noted between right- and left-sided colonic cancer. The independence and influence of different risk factors were tested in multivariable analyses. Among all risk factors, lymph node positivity was the strongest, with a HR of 4.71 (95 per cent c.i. 3.45 to 6.43) in the time period more than 1 to 5 years for a lymph node ratio (LNR) of greater than 0.25 (*Table 3*). Other independent risk factors were: T4 category, rectal cancer, cachexia, and diabetes mellitus. Regarding lifestyle factors, daily smoking was an independent risk factor, whereas a moderate daily intake of alcohol decreased the risk of recurrence. Postoperative adjuvant chemotherapy was given to 46.5 per cent of the patients (colon 52.6 per cent, rectum 35.2 per cent), with a reduction in recurrence risk of 38 per cent. Detailed information on risk factors is shown in *Table 3* and *Table**S1*.

**Table 3:** Risk factors for recurrence within 5 years following curative resection of stage II or III colorectal cancer

	No. of patients	No. of recurrences* (%)	Cumulative incidence of recurrence at 5 years (%)^†^	Time period strata	**Univariable Cox regression** ^†^		Multivariable Cox regression (*n* = 2080)^†^	
					Hazard ratio	*P*	Hazard ratio	*P*
**Smoker **								
No	1909	352 (18.4)	20.4 (18.4, 22.5)		1.00 (reference)		1.00 (reference)	
Yes, occasionally	24	8 (33.3)	33.9 (18.6, 56.6)		1.92 (0.95.3.86)	0.069	1.92 (0.90,4.08)	0.091
Yes, daily	371	87 (23.4)	26.9 (21.7, 33.1)		1.34 (1.06, 1.69)	0.015	1.46 (1.13, 1.89)	0.004
Missing	138	24 (17.4)						
**Alcohol, daily intake**								
None	1541	324 (21.0)	22.4 (20.2, 24.7)		1.00 (reference)		1.00 (reference)	
<3 drinks	505	82 (16.2)	19.9 (15.7, 25.0)		0. 0.76 (0.59, 0.96)	0.024	0.67 (0.52, 0.86)	0.002
≥3 drinks	110	21 (19.1)	25.9 (16.7, 38.7)		91 (0.58, 1.41)	0.658	0.89 (0.56, 1.41)	0.612
Missing	286	44 (15.4)						
**BMI (kg/m^2^)**								
<18.5	56	18 (32.1)	33.3 (22.3, 47.7)		1.83 (1.13, 2.95)	0.014	1.59 (0.96, 2.63)	0.071
18.5–24.9	1088	208 (19.1)	21.4 (18.8, 24.4)		1.00 (reference)		1.00 (reference)	
25.0–29.9	932	179 (19.2)	21.6 (18.6, 24.9)		1.00 (0.82, 1.22)	0.965	1.00 (0.81, 1.24)	0.984
30.0–34.9	286	54 (18.9)	20.3 (15.8, 26.0)		0.97 (0.72, 1.31)	0.848	0.89 (0.63, 1.24)	0.484
≥35.0	77	12 (15.6)	15.9 (0.4, 26.4)		0.77 (0.44, 1.41)	0.417	0.86 (0.48, 1.56)	0.628
Missing	3	0 (0)						
**Diabetes**								
No	2224	416 (18.7)	20.7 (18.8, 22.7)		1.00 (reference)		1.00 (reference)	
Yes	218	55 (25.2)	28.2 (22.0, 35.8)		1.36 (1.02, 1.80)	0.033	1.51 (1.11, 2.06)	0.009
**T category**								
T1–3	2090	362 (17.3)	19.4 (17.5, 21.5)		1.00 (reference)		1.00 (reference)	
T4	347	109 (31.4)	33.5 (28.3, 39.4)		2.01 (1.62, 2.49)	<0.001	2.01 (1.58, 2.56)	<0.001
Missing	5	0 (0)						
**Lymph node ratio^‡^**								
Negative	1287	153 (11.9)	12.9 (11.0, 15.1)	0–1 year	1.00 (reference)		1.00 (reference)	
<0.1	426	72 (16.9)	20.4 (16.0, 25.9)	0–1 year	0.77 (0.46, 1.29)	0.322	1.17 (0.67, 2.03)	0.588
0.1–0.25	351	98 (27.9)	32.4 (26.8, 38.9)	0–1 year	1.21 (0.75, 1.93)	0.434	1.60 (0.95, 2.69)	0.075
>0.25	337	140 (41.5)	43.8 (38.1, 49.9)	0–1 year	3.81 (2.71, 5.35	<0.001	4.69 (3.16, 6.94)	< 0.001
Missing	41	8 (19.5)						
Negative				>1 to 5 years	1.00 (reference)		1.00 (reference)	
<0.1				>1 to 5 years	2.02 (1.43, 2.85)	<0.001	3.14 (2.12, 4.67)	<0.001
0.1–0.25				>1 to 5 years	3.60 (2.64, 4.92)	<0.001	5.32 (3.67, 7.73)	<0.001
>0.25				>1 to 5 years	4.71 (3.45, 6.43)	<0.001	6.39 (4.39, 9.29)	<0.001
**Location**								
Rectum	857	207 (24.2)	27.4 (23.9, 31.2)		1.00 (reference)		1.00 (reference	
Colon	1585	264 (16.7)	18.3 (16.3, 20.5)		0.65 (0.55, 0.78)	<0.001	0.60 (0.49, 0.74)	<0.001
**Adjuvant treatment (postoperative)**								
No	1306	216 (16.5)	18.2 (15.9, 20.7)		1.00 (reference)		1.00 (reference)	
Yes	1136	255 (22.4)	25.0 (22.2, 28.1)		1.40 (1.17, 1.68)	<0.001	0.62 (0.48, 0.80)	0.001

Values in parentheses are *percentages and ^†^95 per cent confidence intervals. The following statistically non-significant or non-confounding risk factors were omitted from the multivariable analysis: age, sex, history of myocardial infarction, pulmonary disease, elective or emergency resection of primary tumour, severe postoperative complication after resection of primary lesion, postoperative blood transfusion. ^‡^Proportional hazards assumption not fulfilled (tested with Schoenfeld’s residuals), so variable fractioned in two time periods.

### Assessment and treatment

Of the 471 patients with recurrences, 418 (88.7 per cent) were assessed by multidisciplinary tumour board (MDT) because of the first recurrence and a total of 253 (53.7 per cent) were assessed as potentially curable. Of these, 225 (47.8 per cent) were finally treated with intent to achieve cure. Among these, 98.2 per cent were assessed in a MDT meeting compared with 80.1 per cent of those not curatively treated (*P* < 0.001). In patients with recurrences confined to one location, 207 of 328 (63.1 per cent) were potentially curatively treated, compared with 17 of 89 (19.1 per cent) with recurrences in two locations, and only 3 of 54 patients (5.6 per cent) with recurrences at three or more sites. The highest rate of potentially curative treatment was noted for liver metastases (112 of 148 patients with liver metastases only). In comparison, 53 of 89 patients (59.6 per cent) with lung metastases only, 15 of 23 (65.2 per cent) with peritoneal metastases only, and 21 of 38 (55.3 per cent) with isolated local recurrences received potentially curative treatment *(Table 4)*. Of the 225 potentially curatively treated patients, 122 (54 per cent) received preoperative or postoperative adjuvant chemotherapy, 99 (44 per cent) had surgery alone, and data were missing for four patients. In the group of patients in whom recurrences were detected by scheduled examinations, 54.5 per cent were considered potentially curatively treated compared with 33.6 per cent of patients whose recurrences were detected by non-scheduled examinations (*P* < 0.001).

**Table 4:** Proportion of curatively treated first recurrences within 5 years in patients primarily radically operated for colorectal cancer stage II and III, stratified by location

Site of metastases	Liver	Lung	Peritoneum	Lymph nodes	Local recurrence	Other
Liver	112 of 148	6 of 23	1 of 5	2 of 11	1 of 6	0 of 1
Lung	6 of 23	53 of 89	0 of 0	0 of 8	2 of 7	0 of 1
Peritoneum	1 of 5	0 of 0	15 of 23	0 of 2	3 of 16	0 of 0
Lymph nodes	2 of 11	0 of 8	0 of 2	6 of 25	2 of 5	0 of 2
Local recurrence	1 of 6	2 of 7	3 of 16	2 of 5	21 of 38	0 of 1
Other	0 of 1	0 of 1	0 of 0	0 of 2	0 of 1	0 of 5
>= 3 sites	1 of 40	1 of 39	0 of 26	0 of 33	0 of 21	1 of 21
Total	123 of 235	62 of 167	19 of 72	10 of 86	29 of 94	1 of 31

### Survival

**Fig. 2:**
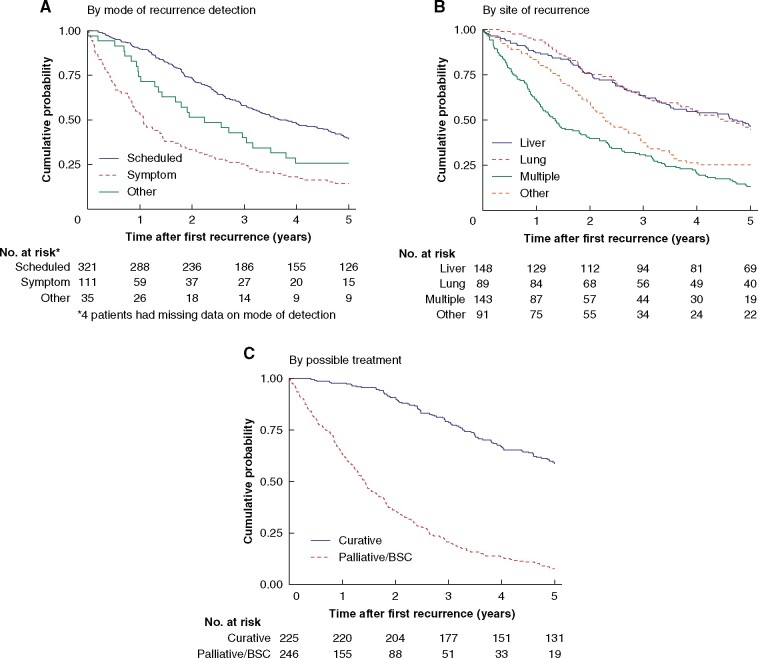
Overall survival from date of first recurrence to death or 5-year follow-up, following radical resection of stage II or III colorectal cancer. Stratified by A) mode of recurrence detection, B) site of recurrence, and C) possible treatment. *Data on mode of detection missing for four patients. BSC, best supportive care.

The 5-year OS rate for all patients with recurrence (calculated from the date of detection) was 32.0 (95 per cent c.i. 27.9 to 36.3) per cent. Sex did not influence survival. Patients with recurrences confined to a single organ had a significantly higher 5-year OS rate (40.2 (95 per cent c.i. 34.9 to 45.5) per cent) than those with two sites (20.4 (12.8 to 29.4) per cent) or multiple sites (2 (0.2 to 8.5) per cent) of recurrence. Patients with recurrences detected by scheduled examinations had a significantly higher 5-year OS rate than those with recurrence detected by symptoms or other reasons for examination (*Fig. 2a*). Survival is shown according to site of recurrence in *Fig 2b*. Patients with recurrences amenable to radical resection had a 7.6-fold higher 5-year OS rate than patients treated with palliative chemotherapy or best supportive care (58.6 *versus* 7.7 per cent) (*Fig. 2c*). HRs for 5-year mortality depending on mode of detection and site of recurrence (adjusted for age, sex, and follow-up regimen) are shown in *Table 5.*

**Table 5:** Five-year overall survival after date of first recurrence in 471 patients following stage II or III curative resection of colorectal cancer

	Proportion of patients who died	5-year OS (%)	Time period strata	Univariable Cox regression		Multivariable Cox regression	
				Hazard ratio	*P*	Hazard ratio	*P*
**Recurrence site**							
Liver	79 of 148	46.6 (38.4, 54.4)		1.00 (reference)		1.00 (reference)	
Lung	49 of 89	44.9 (34.4, 54.9)		1.01 (0.71, 1.44)	0.97	1.24 (0.85, 1.81)	
Multiple sites	124 of 143	13.3 (8.3, 19.4)		2.85 (2.14, 3.78)	<0.001	2.60 (1.94, 3.49)	<0.001
Other sites	68 of 91	25.3 (16.9, 34.5)		1.88 (1.36, 2.60)	<0.001	1.76 (1.25, 2.49)	0.001
**Time from surgery to recurrence (per year)**				0.91 (0.81, 1.02)	0.098	0.84 (0.74, 0.94)	0.020
**Time from surgery to recurrence by group (years)**						Not included	
<1	127 of 180	29.4 (23.0, 36.2)		1.00 (reference)			
1 to <2	105 of 145	27.6 (20.6, 35.0)		1.01 (0.78, 1.31)	0.94		
2 to <3	54 of 86	37.2 (27.1, 47.3)		0.80 (0.58, 1.10)	0.17		
≥3	34 of 60	43.2 (30.5, 55.2)		0.69 (0.47, 1.00)	0.052		
Mode of recurrence detection*							
Scheduled	195 of 321	39.2 (33.9, 44.6)	0–1 year	1.00 (reference)		1.00 (reference)	
Symptom	95 of 111	14.3 (8.6, 21.5)	0–1 year	6.06 (3.91, 9.38)	<0.001	4.60 (2.95, 7.18)	<0.001
Other	26 of 35	25.7 (12.8, 40.8)	0–1 year	2.65 (1.27, 5.54)	0.010	2.40 (1.14, 5.03)	0.013
Scheduled			>1 to 5 years	1.00 (reference)		1.00 (reference)	
Symptom			>1 to 5 years	1.77 (1.26, 2.48)	0.001	1.55 (1.09, 2.19)	0.010
Other			>1 to 5 years	1.37 (0.83, 2.26)	0.214	1.48 (0.89, 2.47)	0.13
**Follow-up regimen**							
Low intensity	166 of 223	25.6 (20.0, 31.4)		1.00 (reference)		1.00 (reference)	
High intensity	154 of 248	37.8 (31.8, 43.9)		0.72 (0.56, 0.89)	0.003	0.80 (0.64, 1.01)	0.058
**Sex**							
M	185 of 271	31.7 (26.2, 37.3)		1.00 (reference)			
F	135 of 200	32.5 (26.1, 39.0)		1.02 (0.82, 1.27)	0.87		
**Age at recurrence (per 10 years)**				1.22 (1.06, 1.40)	0.006	1.29 (1.11, 1.50)	0.001
**Age at recurrence by group (years)**						Not included	
0–59	69 of 118	41.5 (32.6, 50.2)		1.00 (reference)			
60–69	135 of 198	31.8 (25.4, 38.3)		1.32 (0.99, 1.77)	0.058		
≥70	116 of 155	25.2 (18.6, 32.2)		1.65 (1.22, 2.22)	0.001		
**Primary site**							
Rectum	125 of 207	39.6 (32.9, 46.1)		1.00 (reference)		1.00 (reference)	
Colon	195 of 264	26.1 (21.0, 31.6)		1.52 (1.22, 1.92)	<0.001	1.34 (1.05, 1.70)	0.016

Values in parentheses are 95 per cent confidence intervals. OS, overall survival. Statistically non-significant or non-confounding risk factors were omitted from the multivariable analysis. ^*^Proportional hazards assumption not fulfilled (tested with Schoenfeld’s residuals), so variable fractioned in two time periods.

### Ad hoc analyses stratified by follow-up regimen

**Fig. 3:**
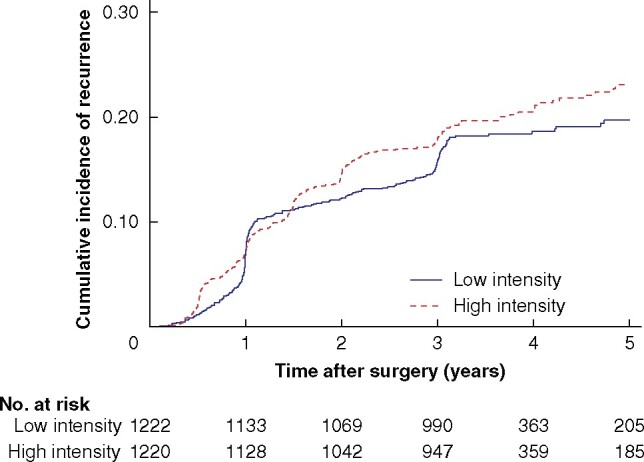
Cumulative incidence of recurrences following radical resection of stage II or III colorectal cancer stratified by follow-up regimen

Of the 471 patients who developed recurrences, a total of 248 were detected in the group randomized to high-intensity follow-up compared with 223 in the group randomized to low-intensity follow-up. The cumulative incidence of recurrence was similar in the high- and low-intensity groups: 23.1 (95 per cent c.i. 20.3 to 26.0) and 19.7 (17.3 to 22.2) per cent respectively. Median time to detection of recurrences was 1.39 years in the high-intensity and 1.03 years in the low-intensity groups (*Fig. 3*). A higher proportion of recurrences were detected by scheduled examinations in the high-intensity group (77.0 *versus* 59.4 per cent; *P* < 0.001).

The proportion of detected recurrences that it was possible to treat potentially curatively was also similar in the two groups (49.2 per cent with high- and 46.2 per cent with low-intensity follow-up). The 5-year OS rate, calculated from the date of detection of recurrences, was better in the high-intensity group: 37.8 (95 per cent c.i. 31.8 to 43.9) *versus* 25.6 (20.0 to 31.4) per cent; however, this difference did not reach statistical significance in the multivariable analysis (*Table 5*).

## Discussion

The recurrence risks of 13.4 per cent in stage II and 30.7 per cent in stage III colorectal cancer are lower than most earlier estimations^3^^,^^4^, but in line with other recent studies^2^^,^^7^^,^^22^, probably reflecting improvements in surgical technique, neoadjuvant treatment, imaging techniques, and structured work-up. Another important factor is the effect of adjuvant chemotherapy as standard care in high-risk stage II and stage III disease. The efficacy of adjuvant treatment in the present study was underlined in the adjusted multivariable analyses, which showed a 38 per cent decreased risk of recurrence. This was slightly higher than previous estimations^7^^,^^23^, which might be due in part to the relatively high proportion of patients receiving this treatment in the present study.

Risk factors associated with recurrence were largely in agreement with previous reports, although the pronounced impact of high LNR (ratio of positive lymph nodes exceeding 0.25) is not widely recognized and merits consideration in the choice of adjuvant therapy. Smoking is a well known risk factor for developing cancer, including colorectal cancer; although described previously as a risk factor for increased risk of recurrence^24^, this has not been reported widely. A finding of interest was that a moderate daily intake of alcohol was associated with a decreased risk of recurrent disease. This requires confirmation in a separate cohort.

It was possible to deliver potentially curative treatment in almost half of the patients with recurrences. Three-quarters of the patients with metastases confined to the liver only were potentially curatively treated, a higher proportion than reported previously^4^^,^^22^^,^^25–27^. Although a greater proportion of patients underwent resection, survival in the operated group was as high, or higher, than in previous reports^4^^,^^19^^,^^22^^,^^25^^,^^28–30^, indicating an absolute survival benefit in this group. Compared with earlier reports^18^^,^^30–32^, potentially curative treatments were also undertaken in higher proportions of patients also for isolated lung metastases (59.6 per cent), isolated peritoneal metastases (65.2 per cent), and isolated local recurrences (55.3 per cent). Among those with recurrences involving lymph nodes, treatment with curative intent was considerably less frequent and possible in only 11 per cent if combined with other sites of recurrence. The high rate of assessment in MDT meetings may have been crucial in achieving these figures. An increased rate of metastases being allocated to resection with curative intent by assessment of organ specialists has been shown for liver metastases^33^ and recurrences of colorectal cancer in general^34^. Improved diagnostics and chemotherapy strategies have also been proven for different diagnoses by MDT assessment^35^.

Patients with recurrences detected by scheduled examinations had a better prognosis than those with recurrences detected by symptoms. This is probably affected by lead time bias as these recurrences are detected earlier but might also be associated with the higher proportion of potentially curatively treated recurrences in this group. If so, it indicates a benefit of the follow-up programme, although examinations were quite limited in both study arms. The high HR (4.60) for mortality associated with recurrences detected by symptoms during the first year indicates that this group consisted of fast-growing aggressive tumours, possibly with a poor chance of long-term survival. Although interval cancer was an independent risk factor for mortality also in the later time period, the impact was much less (HR 1.55). Recurrence of colonic cancer was an independent risk factor for mortality compared with rectal cancer (HR 1.34), possibly related to the proportion of tumours with microsatellite instability (MSI) in the colon, but no data were available on MSI status.

Patients who received potentially curative treatment had a 5-year OS rate of 58.6 per cent, similar to or slightly higher than earlier results^25^^,^^29^^,^^34^^,^^36^, indicating that the increased rate of treatment translated into cure. This is further supported by the 5-year OS rate of 32.0 per cent in the whole group of patients with recurrences. The fact that patients aged over 75 years were not included in the study is likely to have influenced these survival figures. Although outcome was worse for patients whose recurrences were detected within 1 year, the 5-year OS rate in this group was still 29.4 per cent, so early recurrences should not be regarded as a contraindication to treatment with curative intent. These data indicate that structured follow-up, although quite limited, combined with MDT assessment can provide good results in recurrent colorectal cancer.

A limited number of recurrences were detected after the scheduled follow-up time of 3 years, suggesting that this duration of follow-up is sufficient. As expected, recurrences were detected earlier in the high-intensity group during the period of scheduled examinations. This is also reflected by a higher rate of recurrences detected by scheduled examinations in this group. A larger number of recurrences were detected in the high-intensity group after the 3 years of scheduled follow-up, which explains the longer median time to detection of all recurrences in this group. The reason why more recurrences were detected in this group after the period of scheduled examinations is elusive.

The 5-year OS rate after first recurrence was higher in the high-intensity group (calculated from date of detection), probably reflecting lead time bias considering that recurrences were detected earlier on as a result of more frequent examinations. In the multivariable analysis, the HR did not reach statistical significance. Earlier detection might be associated with smaller, treatable recurrences. As more recurrences were detected after 3 years in the group with high-intensity follow-up, this might also have been a factor, as these late recurrences probably have a more favourable prognosis, supported by the multivariable analysis showing a HR of 0.84 per year . In the main study, including the total COLOFOL trial population, no difference in overall or colorectal cancer-specific mortality was noted between the randomization groups, calculated from date of operation of the primary tumour (*P* = 0.43 and *P* = 0.52)^14^.

The major strength of this study is that it was based on a prospectively created data set of recurrences in the framework of a randomized trial with scheduled follow-up, all medical files were scrutinized for detailed data on every recurrence, work-up at diagnosis involved colonoscopy and high-resolution multislice CT of liver and lungs, and a high proportion of recurrences were assessed in MDT meetings. The generalizability is therefore likely to be good, based on an inclusion rate of 56.4 per cent in the main study, and good resemblance between the study population and eligible non-randomized patients according to a drop-out analysis^20^. The cut-off age of 76 years or older for inclusion in the study may also have influenced the proportion of patients treated with curative intent for recurrences.

The main limitation is that scheduled follow-up was limited to 3 years. Thus, recurrences detected between 3 and 5 years after primary surgery were not detected by scheduled examinations but by symptoms or a local follow-up protocol. There is a risk of underestimation of recurrences as a result. As follow-up in the study was 5 years, recurrences that occurred later than 5 years after operation were not registered and the total risk of recurrences might be higher than the 5-year risk presented.

Despite radical primary operation and a high proportion of patients treated with adjuvant chemotherapy, 21.4 per cent of patients with stage II or III colorectal cancer had recurrences. Structured follow-up, although limited, and meticulous MDT review, resulted in a high proportion of recurrences being amenable to potentially curative treatment with subsequent long-term survival.

## Collaborators

Steering Committee: P. Wille-Jørgensen (Bispebjerg University Hospital, Copenhagen, Denmark) (Principal Investigator); E. Horváth-Puhó, S. Laurberg (Aarhus University Hospital, Aarhus, Denmark); the late L. Påhlman (Uppsala Academic Hospital, Uppsala, Sweden); A. Renehan (University of Manchester, Manchester, UK); K. Smedh (Västerås Hospital, Västerås, Sweden); I. Syk (Skåne University Hospital, Malmö, Sweden). Denmark, investigation group: H. Christensen (Aarhus University Hospital, Aarhus); J. D. Nielsen (Aalborg University Hospital, Aalborg); P. Jess (Hillerød Hospital, Hillerød); A. G. Pedersen (Randers Hospital, Randers); M. R. Madsen (Herning Hospital, Herning); P. V. Andersen (Svendborg Hospital, Svendborg); E. Østergaard (Viborg Hospital, Viborg). Sweden, investigation group: P. Hansdotter Andersson (Skåne University Hospital, Malmö); J. Bengtsson (Sahlgrenska Hospital, Gothenburg); M. Bragmark (Danderyd University Hospital, Danderyd); P. Buchwald (Helsingborg Hospital, Helsingborg); M. Egenvall (Karolinska University Hospital Huddinge, Stockholm); P. Farahnak (Södersjukhuset, Stockholm); J. Folkesson (Uppsala Academic Hospital, Uppsala); M. Goldinger (St Görans Hospital, Stockholm); R. Heuman (Mora Hospital, Mora); K. Lindberg (Södertälje Hospital, Södertälje); A. Martling (Karolinska University Hospital Solna, Stockholm); P. Näsvall (Sunderby Hospital, Luleå); J. Ottosson (Kristianstad Hospital, Kristianstad); B. Sandzén (Norrland University Hospital, Umeå). Uruguay, investigation group: C. Barberousse (Maciel University Hospital, Montevideo).

## Supplementary Material

zrab067_Supplementary_Data
