# Supplementary material for: Patterns and resectability of colorectal cancer recurrences: outcome study within the COLOFOL trial
Source: BJS Open. 2021 Jul 26;5(4):zrab067. doi: 10.1093/bjsopen/zrab067 (PMC8311321; doi:10.1093/bjsopen/zrab067)
Supplement: zrab067_Supplementary_Data [file zrab067_supplementary_data.docx]

Supplementary Table 1. Risk factors for recurrence within 5 years following curative operation of colorectal cancer stage II and III, fractioned in two time periods for variables not fulfilling proportional hazards assumption (tested with Schoenfeld’s residuals).

|  | **N** | **Number of  recurrences**  **(%)** | **Cumulative**  **Incidence (%)**  of recurrence  at 5 years (95% CI) | **Number of  competing events**  (%) | **Cumulative**  **incidence**  of competing events at 5 years % (95% CI) | | | **Fractioned**  **in time periods** | **Univariable Cox**  **regression** | | | | **Multivariable Cox**  **regression (N=2080)** | | | |  |
| --- | --- | --- | --- | --- | --- | --- | --- | --- | --- | --- | --- | --- | --- | --- | --- | --- | --- |
|  |  |  |  |  |  |  |  |  | HR (95% CI) | | P | | HR (95% CI) | | P | |  |
| **Age**  <50  50-59  60-64  65-69  70-75 | 176  504  549  615  598 | 31 (17.6)  103 (20.4)  105 (19.1)  118 (19.2)  114 (19.1) | 19.5 (13.6-27.4)  22.8 (18.7-27.6)  21.1 (17.5-25.4)  21.8 (18.2-25.9)  20.7 (17.4-24.6) | 2 (1.1)  7 (1.4)  12 (2.2)  24 (3.9)  33 (5.5) | | 1.2 (0.3-4.5)  1.8 (0.8-4.0)  3.8 (2.0-7.1)  7.8(4.9-12.3)  9.4 (6.5-13.5) | |  | 0.92 (0.62-1.37)  1.08 (0.83-1.42)  0.99 (0.76-1.29)  1.0 (0.77-1.29)  Ref. | | 0.68  0.55  0.94  0.97 | |  | |  | |  |
| **Gender***  Male  Female | 1347  1095 | 271 (20.1)  200 (18.3) | 23.0 (20.4-25.8)  19.2 (16.9-21.9) | 53 (3.9)  25 (2.3) | 7.1 (5.3-9.6)  4.0 (2.5-6.3) | | | **0 – 1 Year**  Male  Female  **>1-5 Years** | Ref.  0.95 (0.71-1.28) | | 0.74 | |  | |  | |  |
|  |  |  |  | | | |  | Male  Female | | Ref.  0.89 (0.70-1.12) | | 0.32 |  | |  |  |  |
| **Smoker**  No  Yes, occasionally  Yes, daily  Missing | 1909  24  371  138 | 352 (18.4)  8 (33.3)  87 (23.4)  24 (17.4) | 20.4 (18.4-22.5)  33.9 (18.6-56.6)  26.9 (21.7-33.1) | 51 (2.7)  1 (4.2)  19 (5.1)  7 (5.1) | 4.8 (3.5-6.5)  4.2 (0.6-26.1)  10.7 (6.4-17.4) | | |  | Ref.  1.92 (0.95.3.86)  1.34 (1.06-1.69) | | 0.069  0.015 | | Ref.  2.01 (0.95-4.28)  1.49 (1.15-1.92) | 0.067  0.001 | |  |  |
| **Alcohol, daily intake** | |  |  |  |  | | |  |  | |  | |  |  | |  |  |
| No  Less than 3 drinks  3 or more drinks  Missing | 1541  505  110  286 | 324 (21.0)  82 (16.2)  21 (19.1)  44 (15.4) | 22.4 (20.2-24.7)  19.9 (15.7-25.0)  25.9 (16.7-38.7) | 41 (2.7)  15 (3.0)  10 (9.1)  12 (4.2) | 4.2 (3.0-5.9)  6.7 (3.7-12.1)  19.2 (10.4-33.8) | | |  | Ref.  0.76 (0.59-0.96)  0.91 (0.58-1.41) | | 0.024  0.66 | | Ref.  0.68 (0.53-0.87)  0.92 (0.58-1.436 | 0.003  0.72 | |  |  |
| **BMI**  <18.5  18.5-24.9  25.0 – 29.9  30 – 34.9  >= 35.0  Missing | 56  1088  932  286  77  3 | 18 (32.1)  208 (19.1)  179 (19.2)  54 (18.9  12 (15.6)  0 (0) | 33.3 (22.3-47.7)  21.4 (18.8-24.4)  21.6 (18.6-24.9)  20.3 (15.8-26.0)  15.9 (0.4-26.4) | 2 (3.6)  34 (3.1)  25 (2.7)  12 (4.2)  4 (4.2)  1 (33.3) | 6.1 (1.4-24.8)  5.0 (3.4-7.3)  5.4 (3.4-8.5)  8.4 (4.6-15.2)  8.9 (3.2-23.8) | | |  | 1.83 (1.13-2.95)  Ref.  1.00 (0.82-1.22)  0.97 (0.72-1.31)  0.77 (0.44-1.41) | | 0.014  0.96  0.85  0.42 | | 1.65 (1.00-2.73)  Ref.  0.98 (0.79-1.22)  0.89 (0.63-1.25)  0.88 (0.49-1.59) | 0.049  0.88  0.50  0.67 | |  |  |
| **Diabetes**  No  Yes | 2224  218 | 416 (18.7)  55 (25.2) | 20.7 (18.8-22.7)  28.2 (22.0-35.8) | 71 (3.2)  7 (3.2) | 5.9 (4.5-7.6)  5.1 (2.3-11.1) | | |  | Ref.  1.36 (1.02-1.80) | | 0.033 | | Ref.  1.53 (1.13-2.09) | 0.007 | |  |  |
| **AMI**  No  Yes | 1665  777 | 319 (19.2)  152 (19.6) | 21.7 (19.5-24.2)  20.7 (17.9-24.0) | 43 (2.6)  35 (4.5) | 4.3 (3.1-6.1)  8.7 (6.0-12.5) | | |  | Ref.  1.02 (0.84-1.23) | | 0.86 | |  |  | |  |  |
| **Pulmonary disease**  No  Yes | 2300  142 | 447 (19.4)  24 (16.9) | 21.5 (19.7-23.6)  18.8 (12.6-27.7) | 67 (2.9)  11 (7.8) | 5.2 (4.0-6.8)  13.2 (7.0-24.1) | | |  | Ref.  0.86 (0.57-1.30) | | 0.48 | |  |  | |  |  |
| **Primary tumour resection** | |  |  |  |  | | |  |  | |  | |  |  | |  |  |
| Elective  Acute | 2314  128 | 438 (18.9)  33 (25.8) | 21.0 (19.2-23.0)  27.4 (20.1-36.6) | 73 (3.2)  5 (3.9) | 5.9 (4.6-7.6)  3.9 (1.7-9.2) | | |  | Ref.  1.43 (1.01-2.04) | | 0.047 | |  |  | |  |  |
| **Severe postoperative complication** | | |  |  |  | | |  |  | |  | |  |  | |  |  |
| No  Yes  Missing | 2192  249  1 | 416 (19.0)  55 (22.1)  0 (0) | 20.8 (19.0-22.8)  26.3 (20.1-34.0) | 67 (3.1)  11 (4.4)  0 (0) | 5.3 (4.0-6.9)  9.9 (5.3-18.1) | | |  | Ref.  1.16 (0.88-1.54) | | 0.29 | |  |  | |  |  |
| **Stage***  II  III | 1315  1127 | 161 (12.2)  310 (27.5) | 13.4 (11.4-15.6)  30.7 (27.6-33.9) | 47 (3.6)  31 (2.8) | 7.0 (5.0-9.5)  4.3 (2.8-6.3) | | | **0-1 Year**  II  III  **>1-5 Years**  II  III | Ref.  1.86 (1.38-2.51)  Ref.  2.91 (2.27-3.73) | | <0.001 | | Not included  Not included |  | |  |  |
| **T-classification**  T1-3  T4  Missing | 2090  347  5 | 362 (17.3)  109 (31.4)  0 (0) | 19.4 (17.5-21.5)  33.5 (28.3-39.4) | 64 (3.1)  14 (4.0)  0 (0) | 5.7 (4.3-7.5)  6.4 (3.6-11.2) | | |  | Ref.  2.01 (1.62-2.49) | | <0.001 | | Ref.  1.96 (1.54-2.50) | <0.001 | |  |  |
| **LNR***  Negative  <0.1  0.1-0.25  >0.25  Missing | 1287  426  351  337  41 | 153 (11.9)  72 (16.9)  98 (27.9)  140 (41.5)  8 (19.5) | 12.9 (11.0-15.1)  20.4 (16.0-25.9)  32.4 (26.8-38.9)  43.8 (38.1-49.9) | 46 (3.6)  11 (2.6)  8 (2.3)  13 (3.9)  0 (0) | 7.2 (5.2-9.9)  4.1 (2.1-7.8)  4.8 (2.2-10.4)  4.7 (2.6-8.4) | | | **0-1 Year**  Negative  <0.1  0.1-0.25  >0.25 | Ref.  0.77 (0.46-1.29)  1.21 (0.75-1.93)  3.81 (2.71-5.35) | | 0.32  0.43  <0.001 | | Ref.*  1.00(0.58-1.72)  1.36 (0.82-2.25)  3.94 (2.70-5.74) | 0.99  0.24  <0.001 | |  |  |
|  |  |  |  |  |  | | | **>1-5Years**  Negative  <0.1  0.1-0.25  >0.25 | Ref.  2.02 (1.43-2.85)  3.60 (2.64-4.92)  4.71 (3.45-6.43) | | <0.001  <0.001  <0.001 | | Ref.  2.67 (1.82-3.92)  4.47 (3.13-6.37)  5.32 (3.73-7.61) | <0.001  <0.001  <0.001 | | | |
| **Location**  Rectum  Colon | 857  1585 | 207 (24.2)  264 (16.7) | 27.4 (23.9-31.2)  18.3 (16.3-20.5) | 25 (2.9)  53 (3.3) | 5.8 (3.7-9.0)  5.8 (4.3-7.8) | | |  | Ref.  0.65 (0.55-0.78) | | <0.001 | | Ref.  0.60 (0.49-0.74) | <0.001 | | | |
| **Adjuvant treatment** | |  |  |  |  | | |  |  | |  | |  |  | | | |
| No  Yes | 988  1454 | 145 (14.7)  326 (22.4) | 16.1 (13.7-18.9)  25.0 (22.5-27.7) | 35 (3.5)  43 (3.0) | 8.2 (5.7-11.6)  4.2 (3.0-5.8) | | |  | Ref.  1.60 (1.32-1.95) | | <0.001 | | Ref.  0.77 (0.60-0.98) | 0.031 | | | |
|  |  |  |  |  |  | | |  |  | |  | |  |  | | | |
| **Postop blood transfusion** | | |  |  |  | | |  |  | |  | |  |  | | | |
| No  Yes | 1990  452 | 376 (18.9)  95 (21.0) | 21.3 (19.3-23.5)  22.0 (18.2-26.4) | 64 (3.2)  14 (3.1) | 5.6 (4.2-7.3)  6.4 (3.7-11.0) | | |  | Ref.  1.12 (0.89-1.40) | | 0.34 | |  |  | | | |

*Proportional hazards assumption not full-filled.
